# Clinical evidence of the role of *Methanobrevibacter smithii* in severe acute malnutrition

**DOI:** 10.1038/s41598-021-84641-8

**Published:** 2021-03-08

**Authors:** Aminata Camara, Salimata Konate, Maryam Tidjani Alou, Aly Kodio, Amadou Hamidou Togo, Sebastien Cortaredona, Bernard Henrissat, Mahamadou Ali Thera, Ogobara K. Doumbo, Didier Raoult, Matthieu Million

**Affiliations:** 1 Aix Marseille Univ, IRD, APHM, MEPHI, 19-21 Boulevard Jean Moulin, 13005 Marseille, France; 2 IHU-Méditerranée Infection, 19-21 Boulevard Jean Moulin, 13005 Marseille, France; 3 Malaria Research and Training Center (MRTC)/Department of Epidemiology of Parasitic Disease/University of Sciences, Techniques and Technologies of Bamako, BP 1805, Bamako, Mali; 4 IRD, APHM, Aix Marseille Univ, VITROME, Marseille, France; 5 Architecture et Fonction des Macromolécules Biologiques, Centre National de La Recherche Scientifique (CNRS) and Aix-Marseille University, Marseille, France; 6 Department of Biological Sciences, King Abdulaziz University, Jeddah, Saudi Arabia

**Keywords:** Microbiology, Molecular biology, Biomarkers, Diseases, Pathogenesis

## Abstract

Gut microbial dysbiosis has been shown to be an instrumental factor in severe acute malnutrition (SAM) and particularly, the absence of *Methanobrevibacter smithii,* a key player in energy harvest. Nevertheless, it remains unknown whether this absence reflects an immaturity or a loss of the microbiota. In order to assess that, we performed a case–control study in Mali using a propensity score weighting approach. The presence of *M. smithii* was tested using quantitative PCR on faeces collected from SAM children at inclusion and at discharge when possible or at day 15 for controls. *M. smithii* was highly significantly associated with the absence of SAM, detected in 40.9% controls but only in 4.2% cases (p < 0.0001). The predictive positive value for detection of *M. smithii* gradually increased with age in controls while decreasing in cases. Among children providing two samples with a negative first sample, no SAM children became positive, while this proportion was 2/4 in controls (p = 0.0015). This data suggests that gut dysbiosis in SAM is not an immaturity but rather features a loss of *M. smithii*. The addition of *M. smithii* as a probiotic may thus represent an important addition to therapeutic approaches to restore gut symbiosis.

## Introduction

Methanogenic archaea play a critical role in host-microbiota mutualism^1^ by removing fermentative dihydrogen (H_2_), which is a central metabolite in overall organic matter degradation^2^. This H_2_ removal allows for a more complete oxidation of substrates, thereby improving energy harvest and the production of key molecules for the host, such as butyrate^3^ and ATP^2,4^. For instance, accumulation of H_2_ inhibits bacterial NADH dehydrogenases, thus reducing the yield of ATP^5,6^. The very low H_2_ utilization threshold of *M. smithii* (10 Pa^6^) compared to that of acetogens makes it the most efficient gut microbe for depleting H_2_ from the gut environment^7^. Moreover, *M. smithii* can alter the specificity and efficiency of digestion of some glycans in animal models^8,9^. These observations suggest a critical clinical relevance of this archaeon for human health.

The critical role of *M. smithii* in energy harvest and glycan digestion regulation has been shown in vitro and in vivo^8,9^. Paradoxically, these earlier publications have neglected the clinical relevance of *M. smithii* in human health, weight regulation and severe acute malnutrition^10–14^. Archaeal methanogens are poorly detected by metagenomics methods. Indeed, specific approaches had to be devised because metagenomics based on the amplification of hypervariable regions of the 16S ribosomal RNA gene was not efficient^15,16^. In a healthy adult European population, we showed that 100% of individuals harbour *M. smithii* in their stool samples, while the second known human gut methanogen, *Methanosphaera stadtmanae*, was rarely found^17^. The ubiquity of *M. smithii* in healthy adults reinforces the idea that this methanogen plays a crucial role in gut microbiota physiology.

Because *M. smithii* has been associated with weight gain and adiposity in animals, it has been speculated that *M. smithii* may promote obesity and that its eradication could treat obesity^8,9^. However, we found that *M. smithii* was associated with normal weight and that obesity was associated with *M. smithii* depletion^18,19^. This has been confirmed by other teams^20,21^. The apparent contradiction between the experimental^8,9^ and clinical^18,19^ results disappears when considering that the presence of *M. smithii* could be associated with normal weight and adiposity and that the relationship between *M. smithii* and body mass index is an inverted U curve. *M. smithii* has also been shown to improve the production of acetate^9^ as well as butyrate, a key molecule for human health^3^, through microbe-microbe interactions^22^. All these unique properties make *M. smithii* one of the best candidates as a marker of healthy digestion and nutritional status.

Severe acute malnutrition is a major public health problem affecting nearly 20 million children under five and causing up to 1 million deaths annually in low- or middle-income countries in Africa and South Asia^22^. It is a severe disease primarily related to inadequate diet. Micronutrients as well as protein-energy deficiencies have been linked to severe acute malnutrition^23–25^. However, cases refractory to a therapeutic diet^26^ and the fact that antibiotics improve mortality rates^26,27^ suggest other instrumental factors, such as gut microbiota dysbiosis. A delay in maturation of the digestive microbiota has been reported^11,13^, suggesting a quantitative developmental abnormality or immaturity. However, this immaturity could be a consequence and not the main feature of gut dysbiosis. A preliminary study conducted using specific quantitative PCR has shown that no malnourished children were positive for *M. smithii* compared to 75% of healthy children^28^. This observation suggests an absence or a loss of *M. smithii* which could contradict the “immaturity hypothesis”. This prompted us to launch a clinical investigation of the key role of *M. smithii* in severe acute malnutrition.

Accordingly, we performed a large case–control study to clarify the association between *M. smithii* and severe acute malnutrition. We particularly investigated whether the findings were consistent with immaturity and whether *M. smithii* remains undetectable after therapeutic renutrition. This hypothesis would suggest that oral intake as a probiotic and/or in organic dairy^29^ of this cultivable archaeon, previously isolated from human milk and colostrum^21^, may be a useful addition in the treatment of severe acute malnutrition.

## Results

### Participant characteristics

**Figure 1:**
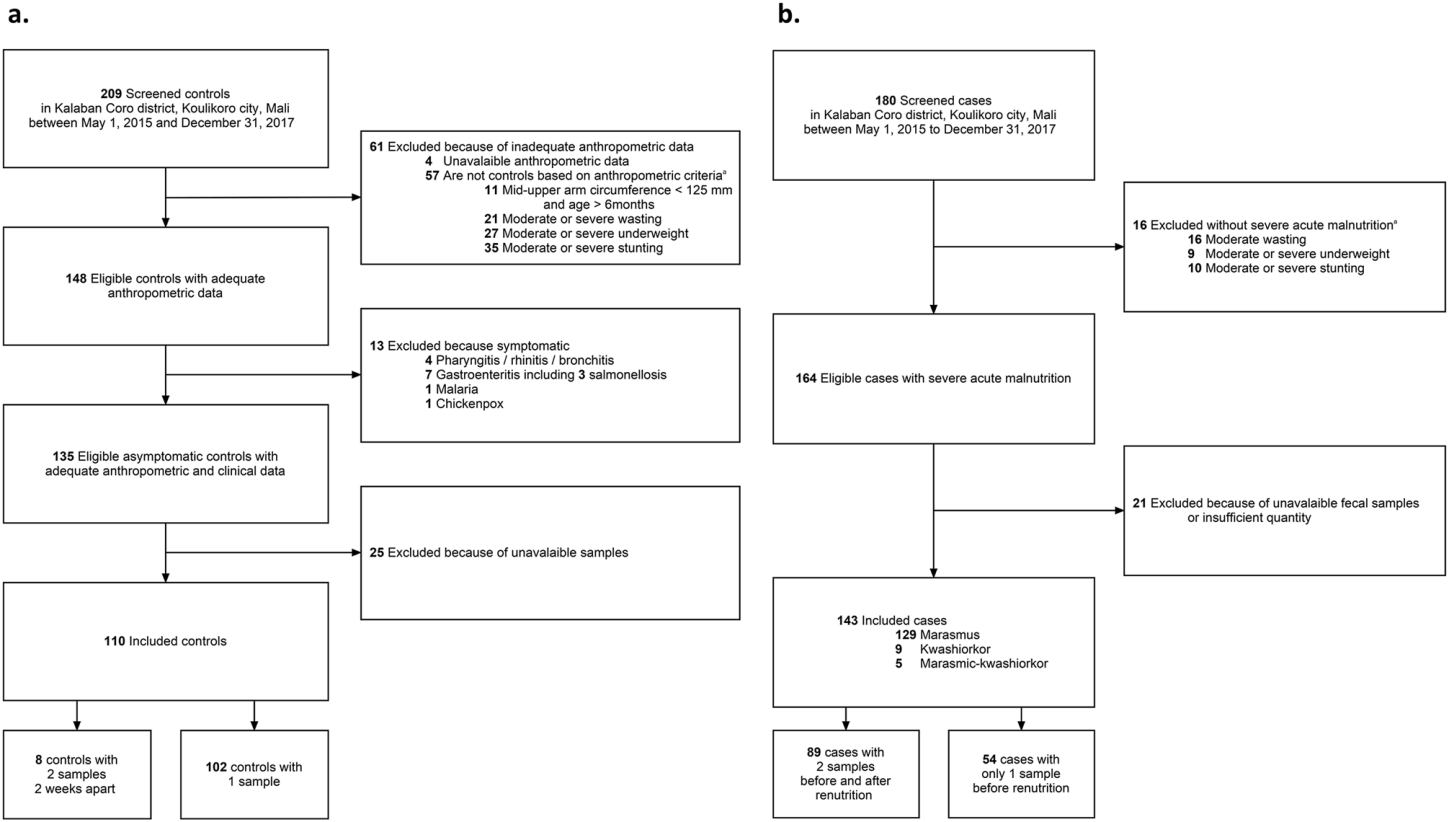
Study flow-chart. (**a**) Case selection, malnourished children are selected based on the WHO severity criterion and before any treatment; the presence of any symptoms or infection was not an exclusion criterion, (**b**) Control selection, healthy children are selected based on WHO standard without symptoms and antibiotics into the last 15 days.

A total of 180 malnourished children were screened. Of these, 16 could not be included because afflicted with moderate acute malnutrition. A total of 164 of them were eligible for study, but only 143 severely malnourished children were ultimately included, as 21 were excluded because of an unavailable or insufficient number of samples provided (Fig. 1a). Of 209 healthy children screened, 61 were excluded outright because they did not meet the adequate anthropometric criteria. Of the 148 eligible controls with adequate anthropometric data, 13 were excluded for the presence of clinical symptoms or diseases such as gastroenteritis, malaria, rhinitis/rhino bronchitis and chickenpox. We excluded 25 others for non-available stool samples. We finally included 110 control children (Fig. 1b).

Our study population comprised 110 healthy controls against 143 severely malnourished infants. Among malnourished children, 129 (90.2%) suffered from marasmus, and the remaining 14 children, less than 10%, had oedema, of whom nine (6.3%) had kwashiorkor and five (3.5%) had marasmic-kwashiorkor (Table 1, Table S1). Malnourished cases and controls were not different regarding age overall (Table S1). Children with marasmus predominantly ranged from 6 to 12 months, while those with kwashiorkor and marasmic-kwashiorkor were older.

**Table 1:** Sociodemographic, nutritional and clinical baseline characteristics—weighted data (n = 253).

Variables	Controls (n = 126)	SAM (n = 127)	Marasmus (n = 111)	Kwashiorkor (n = 7)	Marasmic-Kwashiorkor (n = 9)	p-value^a^ CTL vs SAM	p-value^a^ MRS vs KW vs MRKW
**Demographic characteristics**							
Age (Median)	11 [7–18]	12 [8–17]	11 [7–15]	19 [15–22]	29 [27–32]	0.740	< 0.001
0–6 months	18 (14.3%)	18 (14.2%)	18 (16.2%)	0 (0.0%)	0 (0.0%)		
6–12 months	57 (45.2%)	57 (44.9%)	56 (50.5%)	1 (14.3%)	0 (0.0%)		
12–24 months	37 (29.4%)	38 (29.9%)	30 (27.0%)	6 (85.7%)	1 (11.1%)		
> 24 months	14 (11.1%)	14 (11.0%)	6 (5.4%)	0 (0.0%)	8 (88.9%)	< 0.001	< 0.001
Gender (female)	56 (44.4%)	66 (51.9%)	56 (50.3%)	4 (57.1%)	6 (66.7%)	0.240	0.72
**Context**							
Housewife	100 (79.4%)	70 (55.1%)	65 (58.6%)	3 (42.9%)	2 (22.2%)	< 0.001	0.300
Other children	3.0 [2.0–4.0]	2.0 [1.0–4.0]	2.0 [1.0–4.0]	3.0 [2.0–5.0]	4.0 [4.0–4.0]	0.130	0.001
HIV	N/A	6 (5.0%)	6 (5.7%)	0 (0%)	0 (0%)	–	< 0.001
**Nutritional status**							
Weight (Median [IQR])	9.0 [7.9–10.0]	5.75 [5.1–6.8]	5.5 [5.0–6.6]	8.5 [8.3–8.55]	7.3 [6.7–7.9]	< 0.001	< 0.001
Height (Median [IQR])	73.0 [69.0–80.0]	71.0 [66.0–76.0]	69.0 [65.5–74.0]	76.5 [75.0–77.0]	78.0 [76.0–80.7]	< 0.001	< 0.001
Recumbent	90 (71.4%)	95 (74.8%)	92 (82.9%)	3 (42.9%)	0 (0.0%)	0.550	< 0.001
Œdema	–	16 (12.6%)	–	7 (100%)	9 (100%)	–	
**Anthropometry**							
MUAC^1^ (Median [IQR])	14 [13.5–15.0]	11 [10.0–11.5]	11.0 [10.0–11.3]	13.0 [12.0–13.9]	12.0 [10.5–12.6]	< 0.001	< 0.001
WHZ^2^ (Median [IQR])	− 0.29 [− 1.04 to 0.31]	− 4.05 [− 4.9 to − 3.53]	− 4.13 [− 4.9 to − 3.64]	− 1.56 [− 2.74 to − 1.32]	− 3.72 [− 5.07 to − 3.58]	< 0.001	< 0.001
WAZ^3^ (Median [IQR])	− 0.54 [− 1.15 to 0.17]	− 3.85 [− 4.77 to − 3.13]	− 3.9 [− 4.77 to − 3.26]	− 2.39 [− 2.7 to − 1.49]	− 3.9 [− 5.45 to − 3.75]	< 0.001	< 0.001
HAZ^4^ (Median [IQR])	− 0.71 [− 1.28 to 0.14]	− 2.07 [− 2.99 to − 1.19]	− 2.06 [− 2.98 to − 1.19]	− 1.76 [− 2.64 to − 1.06]	− 2.82 [− 4.43 to − 2.54]	< 0.001	0.180
Wasting	–	123 (97.5%)	111 (100%)	4 (55.6%)	9 (100%)	–	< 0.001
Underweight	–	121 (96.0%)	90 (81.2%)	4 (55.6%)	9 (100%)	–	< 0.001
Stunting	–	67 (52.9%)	57 (51.5%)	4 (44.4%)	7 (77.3%)	–	0.550
**Clinical data**							
Temperature	37.0 [36.7–37.4]	37.4 [36.7–38.1]	37.4 [36.7–38.1]	37.2 [36.5–37.6]	37.5 [36.4–38.0]	< 0.001	0.590
Hyperthermia^5^	1 (0.8%)	14 (11.0%)	14 (12.6%)	0 (0.0%)	0 (0.0%)	< 0.002	0.420
Hypothermia^6^	0 (0.0%)	2 (1.6%)	2 (1.8%)	0 (0.0%)	0 (0.0%)	< 0.001	0.170
Malaria^7^	0 (0.0%)	6 (4.7%)	6 (5.4%)	0 (0.0%)	0 (0.0%)	< 0.001	< 0.001
Digestive symptoms^8^	0 (0.0%)	44 (34.6%)	42 (37.8%)	0 (0.0%)	2 (22.2%)	< 0.001	< 0.001
Diarrhoea	0 (0%)	6.3 (6.3%)	6.3 (7.3%)	0 (0.0%)	0 (0.0%)	0.05	0.9
Gastroenteritis	0 (0%)	29.85 (30.0%)	27.85 (32.4%)	0 (0.0%)	2 (22.8)	0.001	0.6
Emesis	0 (0%)	1.2 (1.2%)	1.2 ‘1.4%)	0 (0.0%)	0 (0.0%)	0.36	0.63
Respiratory symptôms^9^	0 (0.0%)	12 (9.4%)	12 (10.8%)	0 (0.0%)	0 (0.0%)	< 0.001	< 0.001
Candidiasis^10^	0 (0.0%)	12 (9.4%)	11 (9.9%)	1 (14.3%)	0 (0.0%)	< 0.001	< 0.001

*SAM* severe acute malnourished; *CTL* control children; *MRS* marasmus; *KW* kwashiorkor; *MRKW* marasmic-kwashiorkor.

^1^Mid-upper-arm circumference.

^2^Weight-for-height z-score.

^3^Weight-for-age z-score.

^4^Height-for-age z-score.

^5^children with a temperature superior to 38.5.

^6^children with a temperature inferior to 35.

^7^Clinical or biological diagnosis.

^8^Diarrhea, emesis, abdominal pain.

^9^Rhinitis, bronchitis, pneumonia.

^10^Digestive or genital.

^a^Weighted ANOVA for continuous variables and weighted logistic regression for categorical variables. Hypothermia = proportion of individuals are shown.

Controls were asymptomatic by definition and without any known affliction. Fever was more frequent in cases (11.9% (17/143) vs 0.9% (1/110) in controls, p < 0.001, Table S1). Diarrhoea was detected in 6.3% (7/111) of malnourished children, all marasmus cases. Emesis was recorded in only 1% (1/111) of malnourished children. Gastroenteritis, which symptoms include diarrhoea, emesis or both, was diagnosed in 29.7% (33/111) of malnourished children (Table S1).

### *Methanobrevibacter smithii* is lost in severe acute malnutrition

**Figure 2:**
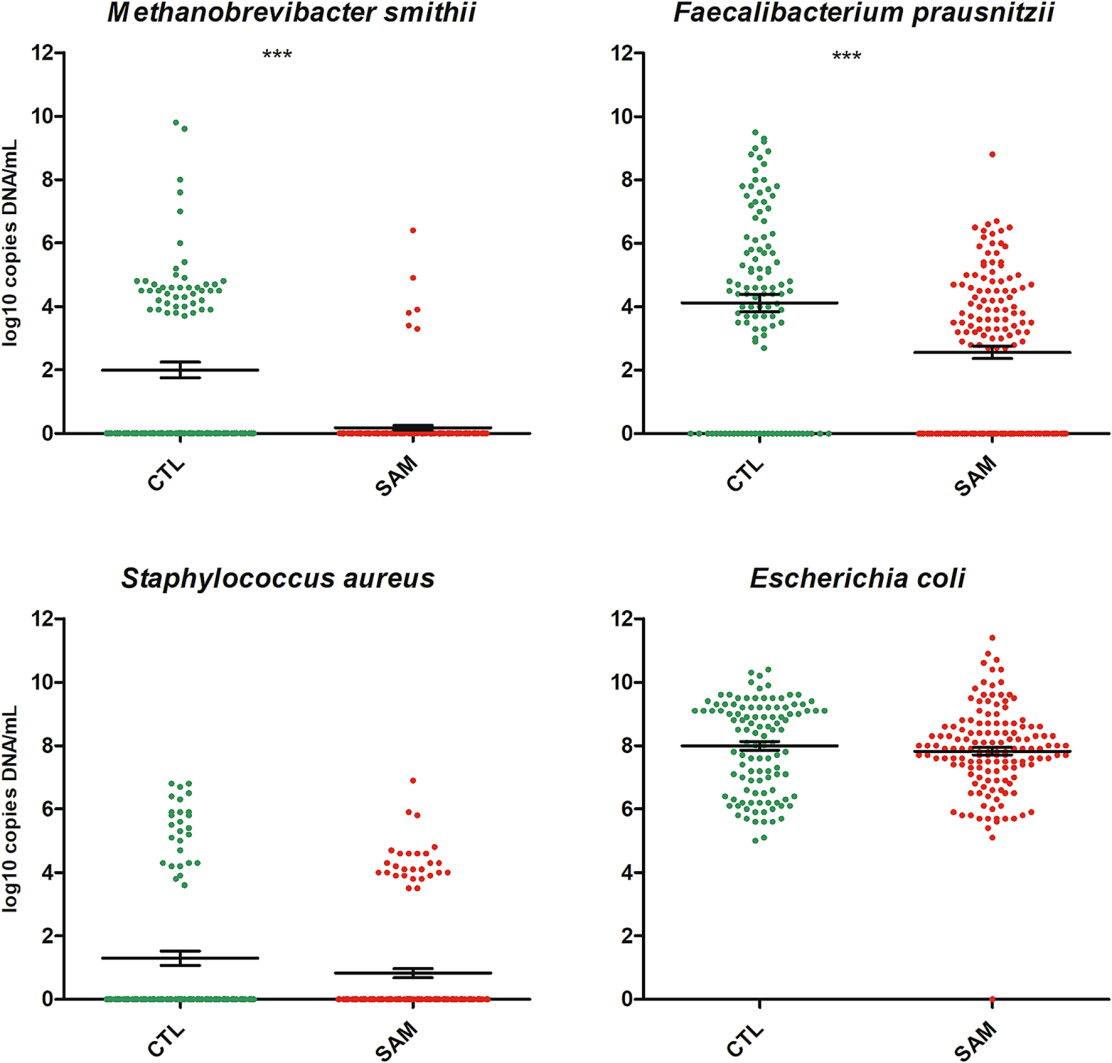
Absolute concentration of *Methanobrevibacter smithii* and other keys species. Species were quantified using real-time PCR in all samples and compared between healthy controls (green points) and children with severe acute malnutrition (red points).

Key species such as *M. smithii, Escherichia coli, Faecalibacterium prausnitzii* and *Staphylococcus aureus* were quantified in severely malnourished children as well as healthy children. The concentration of *M. smithii* was significantly lower in malnourished children as shown in Fig. 2 (0.18 log_10_ DNA copies/ml, SD 0.89 vs 1.99 log_10_ DNA copies/ml, SD 2.56 in healthy children, Mann–Whitney test, p < 0.001). Conversely, the ubiquitous commensal *E. coli* was found in 99.6% (252/253) of the samples at similar mean concentrations in malnourished children and in healthy children (7.82 log_10_ DNA copies/ml, SD 1.39 vs 7.99 log_10_ DNA copies/ml, SD 1.42, Mann–Whitney test, p = 0.19) similarly as *S. aureus* (0.83 log_10_ DNA copies/ml, SD 1.75 in children with severe acute malnutrition vs 1.30 log_10_ DNA copies/ml, SD 2.34 in healthy children, Mann–Whitney test, p = 0.12). *F. prausnitzii* was also found in lower quantities in malnourished children (2.56 log_10_ DNA copies/ml, SD 2.35 vs 4.20 log_10_ DNA copies/ml, SD 2.93 in healthy children, Mann–Whitney test, p < 0.001).

The prevalence of *M. smithii* was dramatically lower in children with severe acute malnutrition compared to controls, 6/143 (4.2%) vs 45/110 (40.9%), respectively, p < 0.001. Strikingly, all positive malnourished children were marasmic (6/129), whereas none (0/14) of the malnourished children with oedema (isolated kwashiorkor or marasmic-kwashiorkor) were positive (Table 2). In order to assess the strength of our results and exclude a possible role of diarrhoea in the reduced prevalence of *M. smithii* in severely malnourished children, children with diarrhoea, emesis or gastroenteritis were excluded. The prevalence in *M. smithii* was still dramatically lower in children with severe acute malnutrition compared to controls, 4/89 (4.9%) vs 45/126 (35.7%), p < 0.001 (Table 3).

**Table 2:** Prevalence of *Methanobrevibacter smithii* detection in children according to age and nutritional status—weighted data (n = 253).

Age	Total (n = 253)	Healthy controls (n = 110)	Severe acute malnutrition (n = 129)	Marasmus (n = 129)	Kwashiorkor (n = 9)	Marasmic-kwashiorkor (n = 5)	p-value^a^
0–6 months	6/36 (16.7%)	6/18 (33.3%)	0/18 (0.0%)	0/18 (0.0%)	–	–	0.060
7–12 months	17/114 (14.9%)	14/57 (24.5%)	3/57 (5.3%)	3/56 (5.3%)	0/1 (0.0%)	–	0.010
13–24 months	14/75 (18.7%)	12/37 (32.4%)	2/38 (5.3%)	2/31 (6.4%)	0/6 (0.0%)	0/1 (0.0%)	0.006
> 24 months	13/28 (46.4%)	13/14 (92.8%)	0/14 (0.0%)	0/6 (0.0%)	0/0 (0.0%)	0/8 (0.0%)	0.002
Overall	50/253 (19.7%)	45/126 (35.7%)	5/127 (3.9%)	5/111 (4.5%)	0/7 (0.0%)	0/9 (0.0%)	< 0.001

^a^Weighted logistic regression comparing the proportion of *M. smithii* positive samples in severely malnourished children compared to healthy controls. When comparing the proportions by age group, there was no significant difference for children with severe acute malnutrition (p = 0.620), but the difference was highly significant for controls (p < 0.001).

**Table 3:** Proportion of detection of *Methanobrevibacter smithii* in children according to age and nutritional status excluding children with diarrhea, emesis and gastroenteritis—weighted data (n = 215).

Age	Total (n = 212)	Healthy controls (n = 126)	Severe acute malnutrition (n = 89)	Marasmus (n = 105)	Kwashiorkor (n = 7)	Marasmic-kwashiorkor (n = 9)	p-value^a^
0–6 months	6/31 (18.7%)	6/18 (33.3%)	0/13 (0.0%)	0/13 (0.0%)	–	–	0.09
7–12 months	16/93 (19.5%)	14/57 (24.6%)	2/36 (6.5%)	2/35 (5.7%)	0/1 (0.0%)	–	0.05
13–24 months	14/69 (20.5%)	12/37 (32.4%)	2/32 (4.9%)	2/25 (8.0%)	0/6 (0.0%)	0/1 (0.0%)	0.01
> 24 months	13/22 (52%)	13/14 (92.9%)	0/8 (0.0%)	0/2 (0.0%)	0/0 (0.0%)	0/6 (0.0%)	0.006
Overall	49/215 (25.1%)	45/126 (35.7%)	4/89 (4.5%)	4/75 (5.3%)	0/7 (0.0%)	0/7 (0.0%)	< 0.0001

^a^Weighted logistic regression comparing the proportion of *M. smithii* positive samples in severely malnourished children compared to healthy controls.

**Figure 3:**
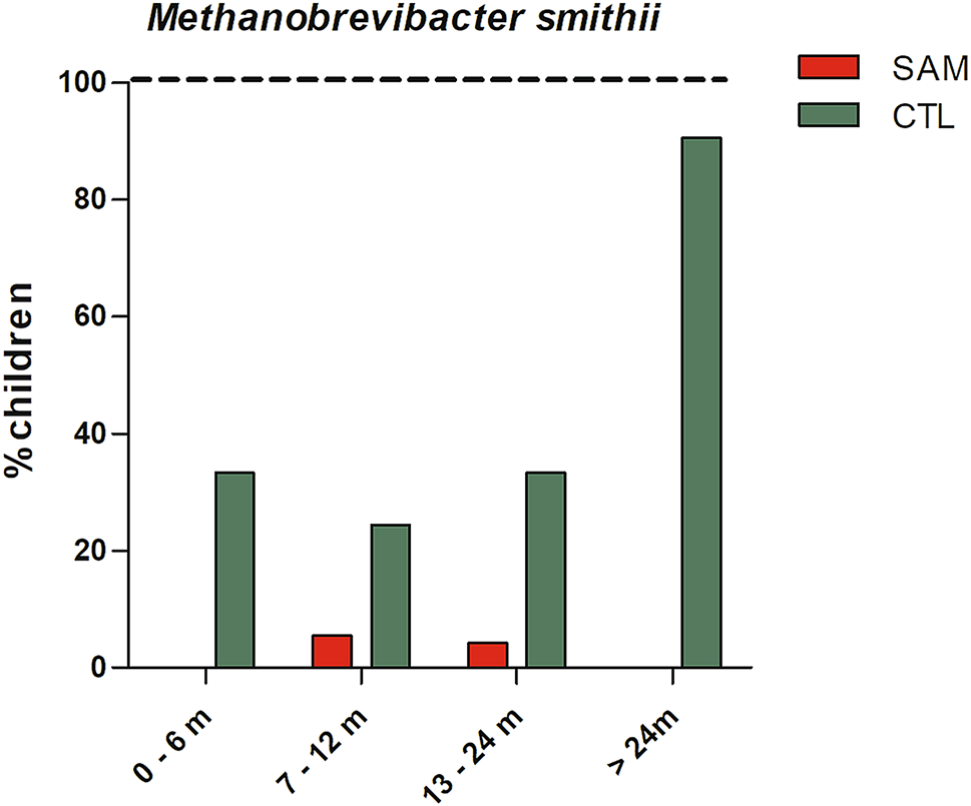
Age group detection of *Methanobrevibacter smithii* in malnourished and healthy children. Proportion of positive children is shown in each age range, green bar represents healthy control and red bar malnourished children.

**Figure 4:**
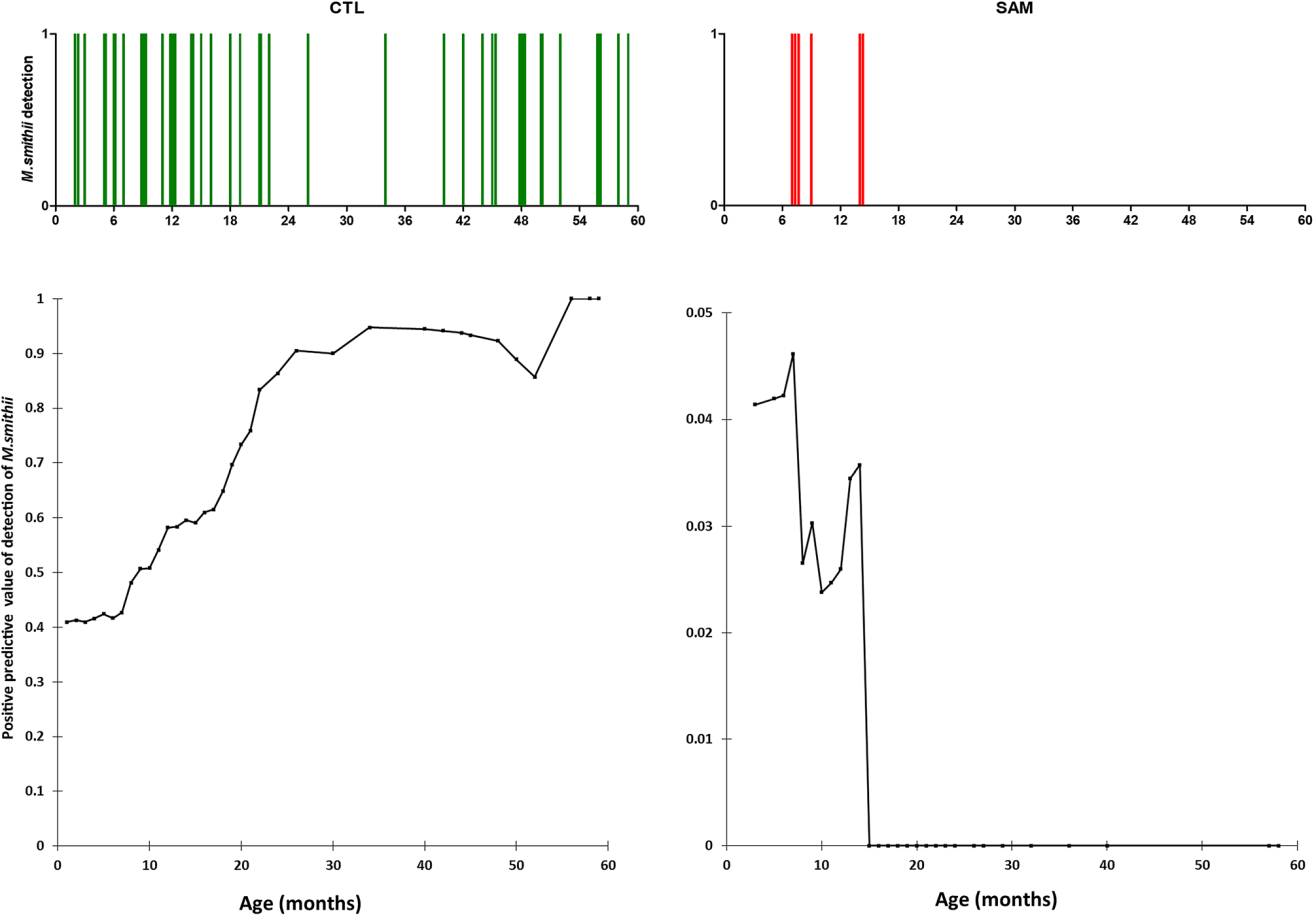
Positive predictive value of *Methanobrevibacter smithii* in healthy and malnourished children according to age. Probability of detection of *M. smithii* relating to age is shown in healthy controls (CTL) and severely malnourished children (SAM); spike bars represent all the individuals from whom *M. smithii* has been detected in each group in the age range from 0 to 59 months. Green spike bars represent healthy controls children in top with the associated predictive value below. Red spike bars represent cases with the associated predictive value below.

**Figure 5:**
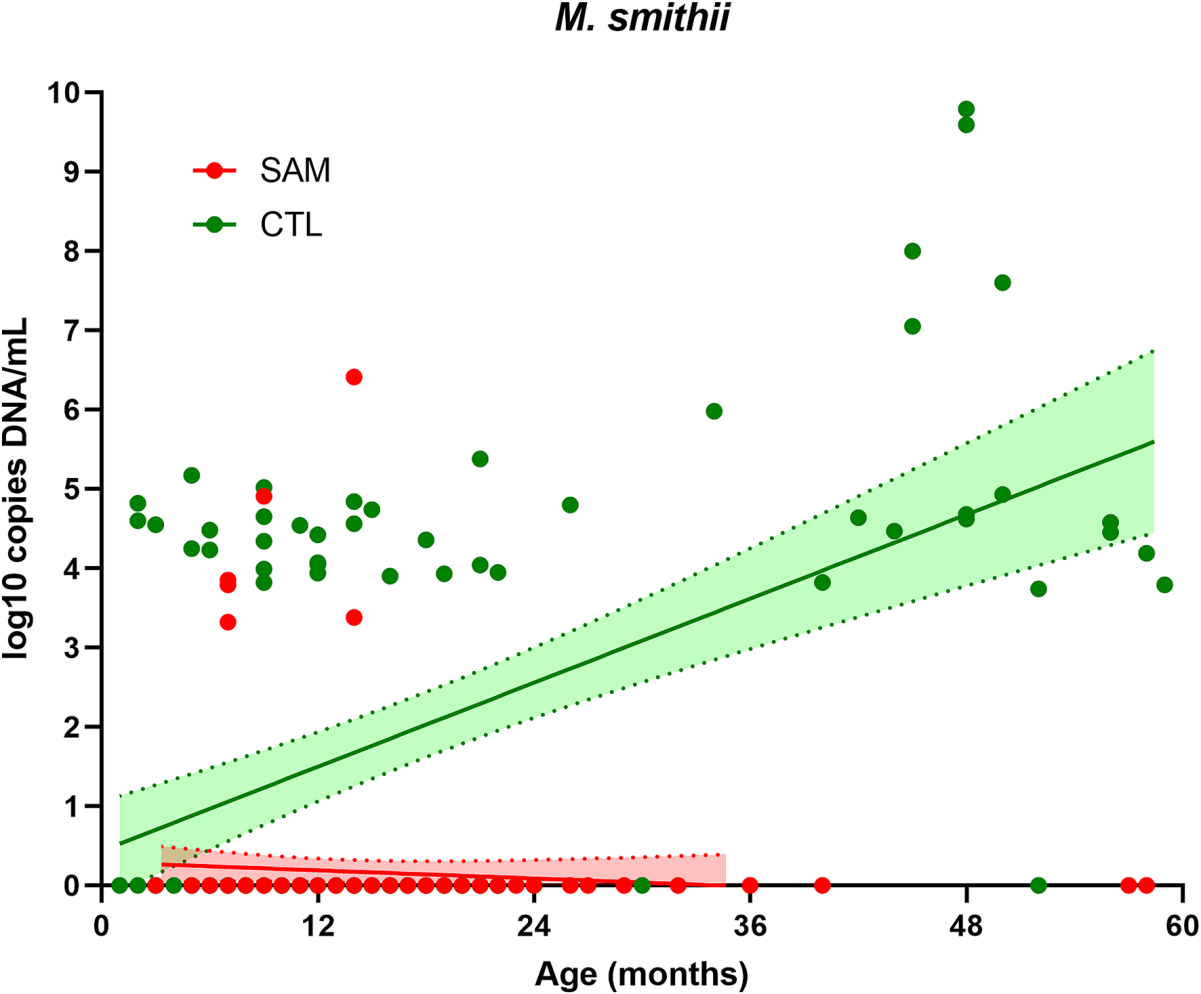
DNA concentration of *Methanobrevibacter smithii* in healthy and severely malnourished children according to age. Linear regression model representing DNA concentrations (log_10_ DNA copies on the Y axis) according to the age of the subject (in months on the X axis). Regression lines are represented separately for each group and its 95% interval confidence range (coefficient of determination r^2^ of 0.29 and 0.006 for controls samples and SAM samples respectively) with the green points and line representing healthy children and the red points and line representing severely malnourished children.

Detection of *M. smithii* increased with age only in controls, while prevalence decreased with age among malnourished children (Figs. 3 and 4). The prevalence rose to 90% in controls but dropped to 0% in severely malnourished children at 24 months (Fig. 3). The predictive positive value of detection of the *M. smithii* curve gradually increased to reach its maximum (all three children aged more than 55 months were positive) before 60 months of age in the healthy controls, unlike that of the malnourished children, which decreased abruptly and was nil after 15 months of age (Fig. 4). Controlling for age and gender, we found that the detection of *M. smithii* was associated with the absence of severe acute malnutrition (OR = 0.06, 95% confidence interval [0.02–0.15], *p* = 1·6 *10^–9)^. We therefore performed a linear regression (Fig. 5) that showed that the concentration of *M. smithii* DNA increased with age only in controls (slope was positive (0.088) and was significantly different from zero in controls (p < 0.001) but negative (-0.0085) in severe acute malnourished children and not different from zero (p = 0.33)), and the difference between the two slopes was significant (p < 0 0.001).

### Therapeutic diet does not reverse the loss of *Methanobrevibacter smithii* in severe acute malnutrition

Among severely malnourished children, therapeutic diet and renutrition were not associated with restoration of *M. smithii* but rather with a loss in the few initially positive children. Indeed, among the 89 severely malnourished children for whom a second sample was available, only one in three children who was initially positive remained positive, while 0/86 children who were initially negative became positive (Table 4). It was more difficult to obtain a second stool sample for the controls. Among 8 healthy children with 2 samples, 2/4 negative samples became positive, while 3/4 positive samples remained positive. The proportion of negative children becoming positive was significantly different according to nutritional status (severe acute malnutrition 0/86 versus healthy children 2/4, two-sided mid-P test, p = 0.001). The proportion of children who became positive or remained positive was significantly lower among severely malnourished children after renutrition (1/89 (1.1%) vs. 5/8 (62.5%), p < 0.001), which suggests that the therapeutic regimen is not able to restore or maintain *M. smithii* in the gut of these children.

**Table 4:** Detection of *Methanobrevibacter smithii* before and after renutrition.

Nutritional status	First sample	Second sample
Severe acute malnutrition	Negative (n = 86)	Negative: 86
		Positive: 0
	Positive (n = 3)	Negative: 2
		Positive: 1
Healthy controls	Negative (n = 4)	Negative: 2
		Positive: 2
	Positive (n = 4)	Negative: 1
		Positive: 3

## Discussion

There is a paradox that the systematic analysis of the presence of *M. smithii* reported here has not been carried out before since this archaeon is a candidate of choice to explain good or bad digestion. Here, we confirmed that *M. smithii*, the main human gut archaeon and a critical human commensal found in virtually all human adults^8,9,17^, is lost (rather than decreased) in severe acute malnutrition. More than 80% of healthy children were positive when older than 20 months, while only 6 of 143 severely malnourished children were positive, all younger than 15 months.

These findings are robust thanks to the large sample size (n = 253) obtained in a different geographical area (Mali) than our previous work, which was located in Niger and Senegal^28^, and to the strict and rigorous selection of malnourished and healthy children, according to clinical data and WHO child growth standards 2006^30^. Selection bias was controlled using a propensity score weighting approach based on the different age groups. All children were sampled during the same period of inclusion to avoid time bias. Stool samples of cases were collected before treatment with antibiotics and renutrition.

Using a DNA extraction protocol optimized for methanogen detection with quantitative real-time polymerase chain reaction, we showed that *M. smithii* was detectable before 24 months but not after 24 months, suggesting a loss of this microbe in children with severe acute malnutrition^31^. Rather than an immaturity of the microbiota, this suggests that the absence of *M. smithii* is a marker of dysbiosis, whether due to obesity or severe acute malnutrition^12,13^, and furthermore supports the association of this archaeon with weight gain in children^32,33^. Control samples were asymptomatic and without any known disease by definition as children selected as controls were excluded in case of gastroenteritis or a moderate or chronic form of malnutrition. In cases, the low prevalence of digestive symptoms such as diarrhoea, emesis as well as that of gastroenteritis could not explain this loss of *M. smithii* as confirmed by the analysis excluding the children suffering from severe acute malnutrition exhibiting the aforementioned symptoms.

We have long believed that *M. smithii* plays an essential role in nutrition. Indeed, Gordon's work in the 2000s showed that the digestion of complex carbohydrates requires the combined action of multiple bacterial strains equipped with a large abundance and diversity of glycosidases^8,9^. Digestion is accompanied by the production of hydrogen, which ultimately inhibits the metabolic activity of these bacteria. This demonstration prompted us to develop a specific method for the extraction and detection of *M. smithii* in faeces^17^. Indeed, 16S rRNA amplifications may miss methanogenic archaea, which have thick walls containing lysozyme-resistant pseudopeptidoglycan and thus require specific DNA extraction procedures^17^. The result of this earlier work was that in a normal French population, 100% of people were methanogen carriers^17^, in contrast with an earlier report that only 30% of the population were carriers^34^.

Since then, we have developed tools for better detection of *M. smithii*. Using these tools, we have noted the absence of *M. smithii* in malnourished patients in Africa^28^, an observation that had escaped other teams working on malnutrition, paradoxically including those of Gordon, whose work inspired our research on *M. smithii*.

In practice, we confirmed the significant absence of *M. smithii* in the faeces of malnourished children. We have previously shown that the source of *M. smithii* in the digestive tract of newborns originated from colostrum and breast milk^21^. Here, we observed a loss of the methanogenic archaea *M. smithii* in the gut microbiota of children with severe acute malnutrition. Severe acute malnutrition is an acute disease with an acute risk of death. Whether this loss is reversible after discharge remains unknown. It is noteworthy that *M. smithii* colonization is associated with organic dairy consumption^29^. Accordingly, organic dairy consumption may help these children recolonize their gut with *M. smithii* after the acute disease. This could be tested in further longitudinal studies with longer observation period post-discharge. Moreover, the acute loss of *M. smithii* may be associated with a sudden collapse of digestion, fermentation and butyrate production which is associated with death in these children^35^. Supplementation with missing microbes^36^, including *M. smithii* by probiotics/organic dairy may be required to prevent death in such a situation.

We believe that it is justified to consider the reintroduction of *M. smithii* in malnourished subjects in the form of a probiotic additive. Breastfeeding or the addition of milk-borne probiotics could be adequate since colonization has been associated with organic dairy consumption^29^. Future milk-based microbiome-directed therapies should investigate the potential benefit of the addition of *M. smithii* to seed the children’s gut and preserve this critical commensal as a key to restoring host-microbial mutualism. In addition, future studies should investigate the possible mechanisms leading to the loss of *M. smithii* and determine whether this loss is the cause of the consequence of other characteristics of the severe acute microbiota associated dysbiosis (loss of aero-intolerant bacterial species among others).

## Methods

### Participants/study design

This case–control study was reported according to the indications of the STROBE statement^37^ (STROBE checklist provided in Table S2) from May 2015 to January 2017 in the peri-urban area of Kalaban Coro located southeast of Bamako in Mali. The cases were severely malnourished children under five years of age and were recruited from the unit of recovery and nutritional education (URENI) of the Kalaban Coro reference health centre. The controls were children of the same age group with no form of acute or chronic illness that can modify the gut microbiota, such as fever, diarrhoea, and/or antibiotic intake, within fifteen days before inclusion.

To test the reversibility of the absence of *M. smithii* in cases and controls, we attempted to obtain a second sample. In the group of malnourished children, this second sample was collected after the acute phase, i.e., after recovery from acute clinical complications (diarrhoea, dehydration, fever, gastroenteritis, respiratory infections), return of appetite with an intake of at least 75% of the daily RUTF ration required for the child and a weight gain of 15 to 20% compared to the entry weight^29^. For control children, a second sample was collected 15 days after the first.

Malnourished infants who did not give stool samples before renutrition were excluded as well as those with acute or chronic illness that may explain their nutritional status. Cases of refusal of consent were also excluded. Case and control children were classified by age range of 0–6 months, 7–12 months, 13–24 months and > 24 months.

### Ethical considerations

The study was started after the approval of the ethics committee of the Faculty of Medicine and Odonto-Stomatology of Bamako, Mali, under the number: N ° 2014/46/CE/FMPOS on May 22, 2014. Informed and signed consent was obtained for all children from their parents or legal guardian in accordance with the Helsinki declaration. Additionally, all experiments were performed in accordance with relevant guidelines and regulations.

### Data sources/measurement/definitions

Anthropometric parameters, including weight, height, mid-upper arm circumference (MUAC) and age, were measured for all participants to determine the nutritional status of the children. We also calculated the weight-for-age, weight-for-height and height-for-age z-scores using the WHO Anthro software (https://www.who.int/childgrowth/software/en/) according to the date of inclusion, gender, date of birth, height measurement recumbent or not, and the presence or absence of oedema. Based on the WHO 2009 severity criterion on acute malnutrition (30), including weight-for-height Z-score (WHZ), weight-for-age Z-score (WAZ), height-for-age Z-score (HAZ), mid-upper arm circumference (MUAC) and the presence of oedema, cases were defined by WHZ <–3 standard deviations (SD), by the presence of nutritional oedema and/or by the MUAC < 115 mm for children over 6 months. Clinical data including temperature (to detect fever), respiratory symptoms and digestive symptoms among which diarrhoea, emesis and gastroenteritis were collected. Moreover, the presence of HIV infection as well as malaria was recorded.

Control children were enlisted in health centres during health monitoring or in the Kalaban Coro health district with anthropometric parameters meeting WHO standards including WAZ > -2 SD, HAZ > -2 SD, WHZ > -2 SD, and MUAC ˃ 125 mm in children older than 6 months and without known disease and who were asymptomatic and without oedema. All the children were screened during the same period and in the same geographical area to address potential sources of bias. A multivariate analysis including age and gender as confounding factors was also performed. In addition, we calculated the size of our sample by referring to the proportion of children positive for *M. smithii* in controls and in children with severe acute malnutrition in our previous study from Niger and Senegal (15/20 in controls vs 0/20 in cases)^28^. According to Fleiss with continuity correction^38^, we needed 8 malnourished compared to 8 controls to be powerful enough (80%) to confirm or infirm the results published in our previous work using a 95% two-sided confidence level^28^. Here, we included 143 cases and 110 controls, i.e. sample sizes well beyond what was required for this study.

### Management of severe acute malnutrition

Management of severe acute malnutrition in Malian URENI (Unités de Récupération et d’Education Nutritionnelle Intensive) consists of three phases, which include, on one hand, nutritional cure with therapeutic milk and ready-to-use therapeutic food (RUTF) and, on the other hand, medical treatment with antibiotics, antiparasitic drugs including antimalarial, and vitamin A supplementation as described by the PECIMA, a programme on the integrated management of severe acute malnutrition (Fig. S1). To avoid therapeutic gut microbiota alteration, faecal samples were collected at admission before administration of any anti-infectious drugs.

### Variables and parameters collected/techniques

#### Gut methanogenic archaea quantification

We performed *M. smithii* detection by targeting the 16S rRNA gene using real-time quantitative polymerase chain reaction using the optimized protocol of *Dridi *et al*.*^17^.

### Real-time quantitative polymerase chain reaction

DNA was extracted manually from 30 mg of feces using the E.Z.N.A. Tissue DNA Kit (Omega Bio-tek, Norcross, GA, USA) according to the manufacturer’s instructions. The total DNA extracted was pure and diluted to the tenth and one-hundredth for real-time quantitative PCR, mainly targeting the 16S rRNA gene of *M. smithii* and *F. prausnitzii*, the sohB gene of *E. coli* and the nucA gene of *S. aureus* (Table S3). All PCRs were performed with a positive control series (plasmids) and negative controls (mix). Specific primers and probe systems were used for amplification (Table S2). Real-time PCR was performed in a total volume of 20 μL, including 10 μL of master mix (Roche Diagnostics GmbH, Mannheim, Germany), 3 μL of distilled water, 0.5 μL of primer Fwd, 0.5 μL of primer Rev, 0.5 μL of probe, 0.5 of uracil-DNA glycosylase (UDG) and 5 μL of DNA. The amplification reactions were performed using the Roche protocol, which consisted of in two minutes at 50 °C, five minutes at 95 °C followed by 40 cycles of five seconds at 95 °C and 30 s at 60 °C and analysed using the CFX96 real-time PCR detection system (Bio-Rad Life Science, Marnes-La-Coquette, France). The real-time PCR results were considered negative in the absence of an amplification curve.

### Statistical methods

The data collected on a questionnaire (supplementary material) were entered in Microsoft Excel and analysed using SPSS software version 20.0 (IBM, Paris, France), SAS 9.4 statistical software (SAS Institute, Cary, NC) and GraphPad Prism 8.0 (GraphPad software, La Jolla, USA). Descriptive statistical analyses were performed for all parameters. The normality test of Shapiro–Wilk was first used for the distribution of quantitative data to apply parametric tests (t tests) or nonparametric tests (Mann–Whitney-Wilcoxon test). The chi-square test was used to test the differences in proportion between groups. All tests were two-tailed. The threshold of significance was set at a value of p ≤ 0.05.

Despite our attempt to recruit controls within the same age group and sex as the cases, controls were still not matched within age categories (p < 0.001, Table S1). In order to control for this confounding factor, we used a propensity score weighting approach on our entire study population. The propensity score was calculated using logistic regression on the age groups. The predicted probabilities from the propensity-score model were used to calculate the stabilized inverse-probability-weighting weights^39^. Associations between groups (cases/controls) and the different variables were then estimated using weighted regressions (normal or logistic depending on the outcome). The positive predictive value (PPV) of *M. smithii* according to age for healthy and malnourished children was calculated. PPV is an estimate of the specificity and sensitivity of a variable, calculated using the following formula: number of true positives/(number of true positives + number of false positives)^40^. We performed linear regression models on the DNA concentration to analyse the dynamics of *M. smithii* relating to age and to compare the speed of expected increase. For this purpose, the slope difference with the horizontal was determined to evaluate to what degree the DNA concentration of *M. smithii* was different from zero in each group and between groups. We performed these calculations on the slope of linear regression following the method described by Zar^41^ and used them in GraphPad Prism 8.0.

## Supplementary Information

Supplementary Information 1.

Supplementary Information 2.
