# Supplementary material for: Clinical evidence of the role of Methanobrevibacter smithii in severe acute malnutrition
Source: Sci Rep. 2021 Mar 8;11:5426. doi: 10.1038/s41598-021-84641-8 (PMC7940396; doi:10.1038/s41598-021-84641-8)
Supplement: Supplementary file 2 — Supplementary Information 2. [file 41598_2021_84641_MOESM2_ESM.pdf]

Clinical Evidence of the Role of *Methanobrevibacter smithii* in Severe Acute Malnutrition

Aminata CAMARA[1,2,3*], Salimata KONATE[1,2,3*], Maryam TIDJANI ALOU PhD[1,2], Aly KODIO[1,2,3], Amadou Hamidou TOGO[1,2,3], Sebastien CORTAREDONA[4], Bernard HENRISSAT PhD DSc[5], Mahamadou Ali THERA MD PhD[3], Ogobara K. DOUMBO, MD PhD[3], Didier RAOULT, MD PhD[1,2,5£], Matthieu MILLION, MD PhD[1,2,£]

[1]Aix Marseille Univ, IRD, APHM, MEPHI, Marseille, France

[2]IHU-Méditerranée Infection, Marseille, France

[3]Malaria Research and Training Center (MRTC)/Department of Epidemiology of Parasitic Disease/University of Sciences, Techniques and Technologies of Bamako, BP 1805, Bamako, Mali

[4]Aix Marseille Univ, IRD, APHM, VITROME, Marseille, France

[5]Architecture et Fonction des Macromolécules Biologiques, Centre National de la Recherche Scientifique (CNRS) and Aix-Marseille University, Marseille, France.

[6]Department of Biological Sciences, King Abdulaziz University, Jeddah, Saudi Arabia

£Corresponding author's e-mail: didier.raoult@gmail.com and matthieumillion@gmail.com

*: Equal contributors

Table S1: Sociodemographic, nutritional and clinical baseline characteristics before weighting

**Variables**	**Controls** (n = 110)	**SAM** (n=143)	**Marasmus** (n = 129)	**Kwashiorkor** (n = 9)	**Marasmic-Kwashiorkor** (n = 5)	**p-value** CTL vs SAM	**p-value** MRS vs KW vs MRKW
**Demographic characteristics**							
Age (Median [IQR])	11.5 [7–21]	12 [8–15]	11 [8–14]	19 [15–22]	29 [27–32]	.62[a]	< .001[b]
0 - 6 months	21 (19.1%)	15 (10.5%)	15 (11.6%)	0 (0.0%)	0 (0.0%)		
6 - 12 months	41 (37.3%)	73 (51.0%)	72 (55.8%)	1 (11.1%)	0 (0.0%)	< .001[c]	< .001 [c]
12 - 24 months	27 (24.5%)	48 (33.6%)	39 (30.2%)	8 (88.9%)	1 (20.0%)		
>24 months	21 (19.1%)	7 (4.9%)	3 (2.3%)	0 (0.0%)	4 (80.0%)		
Gender (female)	48 (43.6%)	75 (52.4%)	67 (51.9%)	5 (55.6%)	3 (60.0%)	.15 [c]	.91 [c]
**Context**							
Housewife	85 (77.3%)	81 (55.5%)	76 (58.9%)	4 (44.4%)	1 (20.0%)	.31 [c]	0.79 [c]
Other children	3.0 [2.0 – 4.0]	2.0 [1.0 – 4.0]	2.0 [1.0 – 4.0]	3.0 [2.0 – 5.0]	4.0 [4.0 – 4.0]	.35[d]	.46[e]
HIV	N/A	6 (4.2%)	6 (4.7%)	0 (0%)	0 (0%)	-	.71
**Nutritional status**							
Weight (Median [IQR])	9.2 [7.5 – 10.3]	5.7 [5.1 – 6.8]	5.5 [5.0 – 6.6]	8.5 [8.3 – 8.55]	7.3 [6.7 – 7.9]	< .001[a]	< .001 [b]
Height (Median [IQR])	73.0 [68.0 – 82.0]	70.5 [66.0 – 75.5]	69.0 [65.5 – 74.0]	76.5 [75.0 – 77.0]	78.0 [76.0 – 80.7]	< .001 [a]	< .001 [b]
Recumbant	73 (66.4%)	114 (79.7%)	110 (85.3%)	4 (44.4%)	0 (0.0%)	.013[e]	< .001[e]
Œdema	-	14 (9.8%)	-	9 (100%)	5 (100%)	< .001[e]	.004[e]
**Anthropometry**							
MUAC[1] (Median [IQR])	14 [13.5 – 15.0]	11 [10.2 - 11.5]	11.0 [10.0 - 11.4]	13.0 [12.0 - 13.9]	12.0 [10.5 - 12.6]	< .001 [a]	< .001 [b]
WHZ[2] (Median [IQR])	-0.3 [-0.96 – 0.31]	-4.05 [-4.87 – -3.53]	-4.1 [-4.9 – -3.64]	-1.56 [-2.74 – -1.32]	-3.72 [-5.07 – -3.58]	< .001 [a]	< .001 [b]
WAZ[3] (Median [IQR])	-0.55 [-1.15 – 0.18]	-3.85 [-4.59 – -3.13]	-3.9 [-4.68 – -3.29]	-2.39 [-2.7 – -1.49]	-3.9 [-5.45 – -3.75]	< .001 [a]	< .001 [b]
HAZ[4] (Median [IQR])	-0.69 [-1.27 – 0.01]	-2.04 [-2.88 – -1.19]	-2.02 [-2.86 – -1.2]	-1.76 [-2.64 – -1.06]	-2.82 [-4.43 – -2.54]	< .001 [a]	.22 [b]
Wasting	-	137 (95.8%)	129 (100%)	2 (22.2%)	5 (100%)	-	< .001 [c]
Underweight	-	138 (96.5%)	128 (99.2%)	5 (55.6%)	5 (100%)	-	< .001 [c]
Stunting	-	73 (51.0%)	65 (50.4%)	4 (44.4%)	4 (80%)	-	.40 [c]
**Clinical data**							
Temperature	37.0 [36.7 – 37.4]	37.4 [36.7 – 38.1]	37.4 [36.7 – 38.1]	37.2 [36.5 – 37.6]	37.5 [36.4 – 38.0]	< .001 [d]	0.85[e]

Hyperthermia[5]	1 (0.9%)	17 (11.9%)	17 (13.2%)	0 (0.0%)	0 (0.0%)	<.001 [c]	.42 [c]
Hypothermia[6]	0 (0.0%)	2 (1.4%)	2 (1.6%)	0 (0.0%)	0 (0.0%)	.28 [c]	.17 [c]
Anorexia	0 (0.0%)	22 (15.0%)	19 (14.4%)	1 (11.1%)	2 (40.0%)	< .001 [c]	.44 [c]
Malaria[7]	0 (0.0%)	7 (4.9%)	7 (5.4%)	0 (0.0%)	0 (0.0%)	< .001 [c]	.004 [c]
**Digestive symptoms[8]**	0 (0.0%)	48 (33.6%)	47 (36.4%)	0 (0.0%)	1 (20.0%)	< .001 [c]	.032 [c]
Diarrhoea	-	7 (6.3%) [f]	7 (7.0%) [f]	0 (0.0%)	0 (0.0%)		0.9
Emesis	-	1 (0.9%) [f]	1 (1.0%) [f]	0 (0.0%)	0 (0.0%)		0.5
Gastroenteritis	-	33 (29.7%) [f]	32 (32.0%) [f]	0 (0.0%)	1 (20.0%)		0.5
Respiratory symptôms[9]	0 (0.0%)	14 (9.8%)	14 (10.9%)	0 (0.0%)	0 (0.0%)	< .001 [c]	.008 [c]
Candidiasis[10]	0 (0.0%)	14 (9.8%)	13 (10.1%)	1 (11.1%)	0 (0.0%)	< .001 [c]	.50 [c]

SAM= Severe acute malnourished; CTL= control children; MRS= marasmus; KW= kwashiorkor; MRKW= marasmic-kwashiorkor [1]=Mid-upper-arm circumference ; [2]=Weight-for-height z-score ; [3]=Weight-for-age z-score ; [4]=Height-for-age z-score ; [5]= children with a temperature superior to 38.5; [6]= children with a temperature inferior to 35; [7]= clinical or biological diagnosis; [8]=diarrhea, emesis, gastroenteritis ; [9]=rhinitis, bronchitis, pneumonia; [10]=digestive or genital ; [a]= Mann-Whitney test ; [b]= Kruskall-Wallis test; [c]=khi-square test; [d]=t-test student ; [e]=one-way ANOVA ; [f]= samples with unavailable data were excluded (n=111). Hypothermia= proportion of are shown

Table S2: STROBE Statement

	Item No	Recommendation	Page No
**Title and abstract**	1	(*a*) Indicate the study's design with a commonly used term in the title or the abstract	1
		(*b*) Provide in the abstract an informative and balanced summary of what was done and what was found	2
**Introduction**			
Background/rationale	2	Explain the scientific background and rationale for the investigation being reported	3-4
Objectives	3	State specific objectives, including any prespecified hypotheses	4
**Methods**			
Study design	4	Present key elements of study design early in the paper	4
Setting	5	Describe the setting, locations, and relevant dates, including periods of recruitment, exposure, follow-up, and data collection	5
Participants	6	(*a*) Give the eligibility criteria, and the sources and methods of case ascertainment and control selection. Give the rationale for the choice of cases and controls	5-6
		(*b*) For matched studies, give matching criteria and the number of controls per case	5-6
Variables	7	Clearly define all outcomes, exposures, predictors, potential confounders, and effect modifiers. Give diagnostic criteria, if applicable	6
Data sources/ measurement	8*	For each variable of interest, give sources of data and details of methods of assessment (measurement). Describe comparability of assessment methods if there is more than one group	6-7
Bias	9	Describe any efforts to address potential sources of bias	5-7
Study size	10	Explain how the study size was arrived at	6
Quantitative variables	11	Explain how quantitative variables were handled in the analyses. If applicable, describe which groupings were chosen and why	7
Statistical methods	12	(*a*) Describe all statistical methods, including those used to control for confounding	8
		(*b*) Describe any methods used to examine subgroups and interactions	8
		(*c*) Explain how missing data were addressed	
		(*d*) If applicable, explain how matching of cases and controls was addressed	8
		(*e*) Describe any sensitivity analyses	

**Results**			
Participants	13*	(a) Report numbers of individuals at each stage of study—eg numbers potentially eligible, examined for eligibility, confirmed eligible, included in the study, completing follow-up, and analysed	8
		(b) Give reasons for non-participation at each stage	
		(c) Consider use of a flow diagram	
Descriptive data	14*	(a) Give characteristics of study participants (eg demographic, clinical, social) and information on exposures and potential confounders	15
		(b) Indicate number of participants with missing data for each variable of interest	
Outcome data	15*	Report numbers in each exposure category, or summary measures of exposure	

Main results	16	(*a*) Give unadjusted estimates and, if applicable, confounder-adjusted estimates and their precision (eg, 95% confidence interval). Make clear which confounders were adjusted for and why they were included	
		(*b*) Report category boundaries when continuous variables were categorized	
		(*c*) If relevant, consider translating estimates of relative risk into absolute risk for a meaningful time period	
Other analyses	17	Report other analyses done—eg analyses of subgroups and interactions, and sensitivity analyses	
**Discussion**			
Key results	18	Summarise key results with reference to study objectives	11
Limitations	19	Discuss limitations of the study, taking into account sources of potential bias or imprecision. Discuss both direction and magnitude of any potential bias	12
Interpretation	20	Give a cautious overall interpretation of results considering objectives, limitations, multiplicity of analyses, results from similar studies, and other relevant evidence	12-13
Generalisability	21	Discuss the generalisability (external validity) of the study results	
**Other information**			
Funding	22	Give the source of funding and the role of the funders for the present study and, if applicable, for the original study on which the present article is based	14

*Give information separately for cases and controls.

Note: An Explanation and Elaboration article discusses each checklist item and gives methodological background and published examples of transparent reporting. The STROBE checklist is best used in conjunction with this article (freely available on the Web sites of

PLoS Medicine at http://www.plosmedicine.org/, Annals of Internal Medicine at http://www.annals.org/, and Epidemiology at http://www.epidem.com/). Information on the STROBE Initiative is available at http://www.strobe-statement.org.

Table S3: Primers and probes used for Real-Time PCR

Organism	Name	Nucleotides sequences	Targeted genes
Methanobrevibacter smithii	16S_740_F	CCGGGTATCTAATCCGGTTC	16S rRNA
	16S_862_R	CTCCCAGGGTAGAGGTGAAA	
	16S_P	6FAM- CCGTCAGAATCGTTCCAGTCAG -MGB	
Faecalibacterium prausnitzii	16S_F	CCCGGCATCGGGTAGAG	16S rRNA
	16S_R	GGACGCGAGGCCATCTC	
	16S_P	6FAM- AAAAGGAGCAATCCGCT -MGB	
Escherichia coli	SohB_F	AGAAATTCCGCGAAGAGYTG	sohB
	SohB_R	TACCAGTGTTCACCCGTTGC	
	SohB_P	6FAM- TGAAGCGTATGCGTCCGTCTCTGG	
Staphylococcus aureus	NucA_F2	GTTGTGGATGGTGATACATTTATTGC	nucA
	NucA_R2	CCAAATGGTTGTACAGGCGTATTC	
	NucA_P2	6FAM- AGGCTTATAGGGGTTGATACGCCAGAAACGG	

Figure S1: Summary of the management of Severe Acute Malnutrition

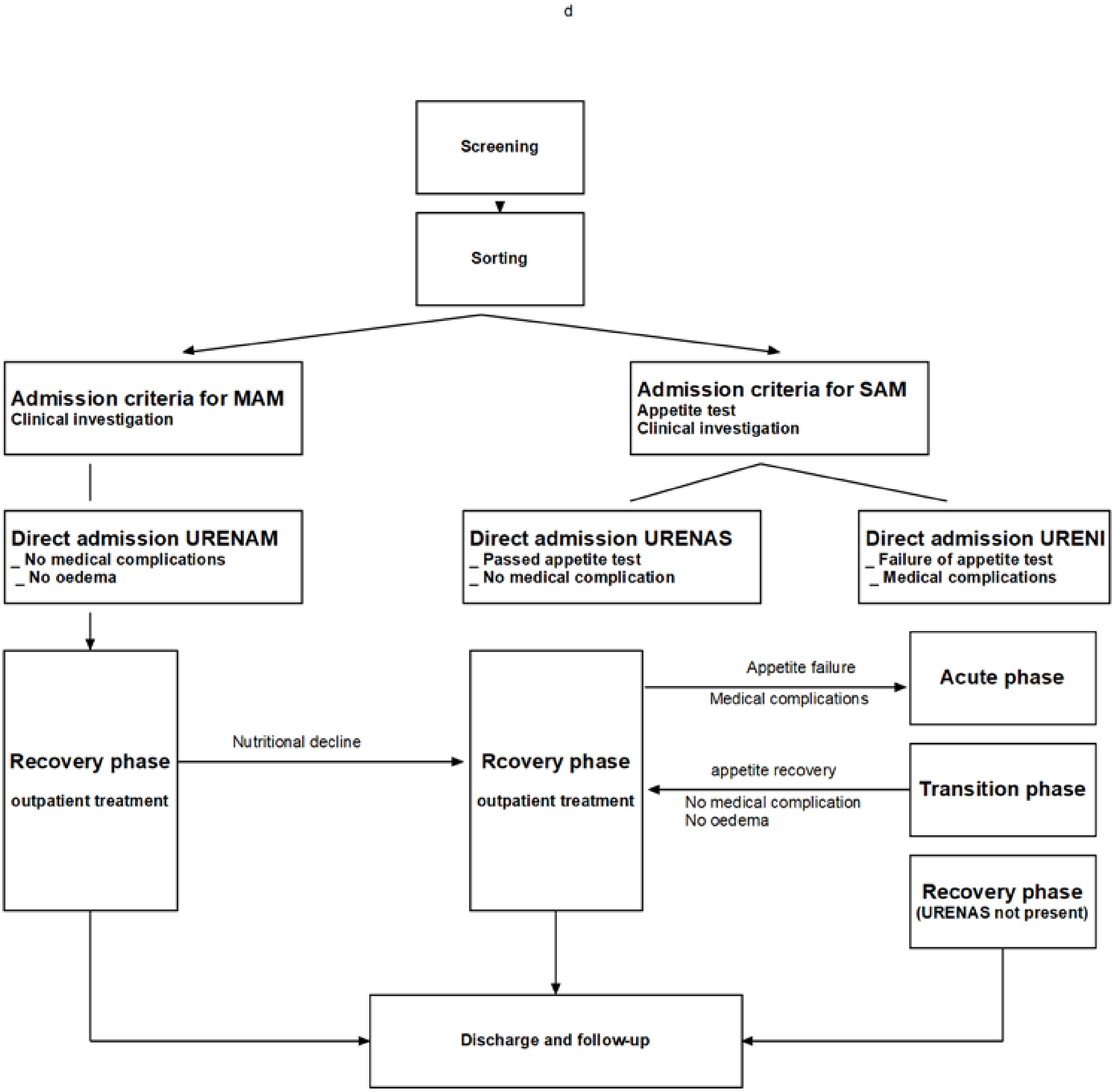

Supplementary Material: Sociodemographic and clinical questionnaire of the study

Ministry of Education Républic of MALI

Malaria Research and Training Center (MRTC)/ Department of Epidemiology of Parasitic Disease/ University of Sciences, Techniques and Technologies of Bamako, BP 1805, Bamako, Mali

Research Protocol

Title : Dynamic and gut microbiote composition in severe acute malnutrition in Mali : Control case report form

Survey

1- Identification and sociodemographics variables

2- Date of'inclusion/ / / / Investigator's name------------------

ID number/ / /

Name-- Surname--

Age / / months Sex F /----/ M/------/

Ethnic--- Résidence -------------------------------------

Father's profession--------------------------------- Mother's profession------------------------

Number of children-----------

Delivery route---

Breastfeeding only /-----/ Breastfeeding + others food/----/ 1=Yes, 2=No

if yes âge-------- in months

if yes --

Antibiotics intake /-----/ 1= Yes; 2= No

If yes which food---; **Date /-----/------/------**

Acute illness :1= Fever, 2= Malaria, 3= Tyhus, 4= Gastroenteric diseases, 5=HIV, 6=others--

Chronic illness/-------/ 1= Yes, 2= No

3-Variables cliniques : 1= Yes; No

Température (Celsius) : /-----. ----/ Weight------Kg ; Height--------cm, MUAC-----------cm,

WHZ<- 2 SD/-----/

WAZ<- 2 SD/-----/

HAZ<-2 SD/------/

Nutritinnal oedema/-----/

Stool collection---------

Date of collection /------/------/---------/

4-Conclusion

Inclued /-------/ **Exclued /--------/**
